# Activation of GABA_B_R Attenuates Intestinal Inflammation by Reducing Oxidative Stress through Modulating the TLR4/MyD88/NLRP3 Pathway and Gut Microbiota Abundance

**DOI:** 10.3390/antiox13091141

**Published:** 2024-09-21

**Authors:** Ziteng Deng, Dan Li, Lu Wang, Jing Lan, Jiaqi Wang, Yunfei Ma

**Affiliations:** State Key Laboratory of Veterinary Public Health and Safety, College of Veterinary Medicine, China Agricultural University, Beijing 100193, China; ztdeng28@cau.edu.cn (Z.D.); lidan@cau.edu.cn (D.L.); s20233051058@cau.edu.cn (L.W.); b20213050382@cau.edu.cn (J.L.); b20233050442@cau.edu.cn (J.W.)

**Keywords:** intestinal inflammation, oxidative stress, GABA_B_R, IPEC-J2 cells, intestinal flora

## Abstract

Oxidative stress emerges as a prominent factor in the onset and progression of intestinal inflammation, primarily due to its critical role in damaging cells and tissues. GABAergic signaling is important in the occurrence and development of various intestinal disorders, yet its effect on oxidative stress remains unclear. We attempted to assess whether GABAergic signaling participated in the regulation of oxidative stress during enteritis. The results showed that lipopolysaccharide (LPS) significantly decreased γ-aminobutyric acid (GABA) levels in the ileal tissues of mice. Interestingly, the application of GABA significantly repressed the shedding of intestinal mucosal epithelial cells and inflammatory cell infiltration, inhibited the expressions of proinflammatory factors, including granulocyte colony-stimulating factor and granulocyte-macrophage colony stimulating factor, and enhanced the levels of anti-inflammatory cytokines interleukin (IL)-4 and IL-10, indicating that GABA could alleviate enteritis in mice. This observation was further supported by transcriptome sequencing, revealing a total of 271 differentially expressed genes, which exhibited a marked enrichment of inflammatory and immune-related pathways, alongside a prominent enhancement of GABA B receptor (GABA_B_R) signaling following GABA administration. Effectively, Baclofen pretreatment alleviated intestinal mucosal damage in LPS-induced mice, suppressed proinflammatory cytokines IL-1β, IL-6, and tumor necrosis factor alpha expressions, and boosted total antioxidant capacity, superoxide dismutase (SOD), and glutathione (GSH) levels. Moreover, Baclofen notably enhanced the viability of LPS-stimulated IPEC-J2 cells, contracted the proinflammatory secretion factors, and reinforced SOD, GSH, and catalase levels, emphasizing the anti-inflammatory and antioxidant effects associated with GABA_B_R activation. Mechanistically, Baclofen restrained the mRNA and protein levels of toll-like receptor 4 (TLR4), myeloid differentiation factor 88 (MyD88), nucleotide-binding oligomerization domain, leucine-rich repeat and pyrin domain-containing 3 (NLRP3), and inducible nitric oxide synthase, while elevating nuclear factor erythroid 2-related factor 2 and heme oxygenase-1 in both mice and IPEC-J2 cells, indicating that activating GABA_B_R strengthened antioxidant abilities by interrupting the TLR4/MyD88/NLRP3 pathway. Furthermore, 16S rDNA analysis demonstrated that Baclofen increased the relative abundance of probiotic, particularly *Lactobacillus*, renowned for its antioxidant properties, while reducing the relative richness of harmful bacteria, predominantly *Enterobacteriaceae*, suggesting that GABA_B_R signaling may have contributed to reversing intestinal flora imbalances to relieve oxidative stress in LPS-induced mice. Our study identified previously unappreciated roles for GABA_B_R signaling in constricting oxidative stress to attenuate enteritis, thus offering novel insights for the treatment of intestinal inflammation.

## 1. Introduction

Since the gut serves as the primary site for nutrient digestion and absorption, intestinal health plays a crucial role in safeguarding the overall health of humans and animals [1]. Intestinal epithelial cells are widely acknowledged as the primary defense system of the host, functioning as a robust barrier to protect against various infectious and non-infectious agents, thus serving as an important role in maintaining the integrity of the intestinal mucosal barrier [2]. The release of lipopolysaccharide (LPS) by *Escherichia coli* has emerged as a significant pathogenic factor that induces intestinal inflammation [3,4]. By stimulating the secretion of inflammatory cytokines, chemokines, and colony-stimulating factors, LPS undermines intestinal epithelial and goblet cells, disrupts the intestinal microbiota, and ultimately weakens the intestinal tissue structure and mucosal barrier [5,6]. As a result, this impairs the capacity of the intestine to absorb and metabolize external nutrients. However, the pathogenesis of intestinal inflammation is highly intricate, and currently, there lacks a particularly effective therapeutic drug to treat it. Hence, it becomes crucial to delve deeper into the pathogenic mechanisms that underlie intestinal inflammatory disease.

Oxidative stress emerges as a crucial trigger for numerous intestinal disorders and holds a significant position in propelling the advancement of intestinal inflammation [7]. Numerous reports show that oxidative stress possesses the potential to inflict damage on the gastrointestinal mucosal layer, thereby clearing the path for bacterial invasion [8]. Subsequently, this invasion ignites an exaggerated immune response within the body, culminating in inflammation. The endogenous antioxidant system plays immense significance in alleviating the damage caused by oxidative stress, which is frequently encountered in inflammatory bowel diseases [9]. Nuclear factor erythroid 2-related factor 2 (NRF2) is a prominent regulator of the antioxidant defense system [10,11]. It has been widely demonstrated that activating NRF2 can amplify the expression of antioxidant enzymes, such as HO-1 and NAD (P) Hquinone oxidoreductase-1 (NQO1) [12,13]. This activation contributes to an elevation in antioxidant molecules, including superoxide dismutase (SOD), glutathione (GSH), glutathione peroxidase (GPx), and catalase (CAT) levels, thereby mitigating intestinal damage. Thus, it is believed that an increase in antioxidant capacity has the potential to mitigate intestinal inflammation and suggests an underlying therapeutic approach for enteritis.

γ-aminobutyric acid (GABA) functions as the foremost inhibitory neurotransmitter in the central nervous system and is prevalent throughout the gastrointestinal tract. Research shows that enteric neurons constitute the main source of gastrointestinal GABA, with immune cells, specifically lymphocytes and macrophages, also playing a role in its synthesis and release [14,15]. Furthermore, evidence has shown that intestinal microorganisms, including *Bacteroides*, *Lactobacillus*, *Bifidobacterium*, and *Parabacteroides*, possess the capability to produce GABA [16]. The GABAergic signaling system is intricately connected to the onset and advancement of diverse gastrointestinal disorders. Pretreatment of HT-29 cells with GABA notably constricts the overexpression of IL-1β, IL-8, and TNF-α provoked by LPS exposure [17]. Conversely, employing bicuculline methiodide to hinder GABA_A_R elicits a contrasting effect to that of GABA, indicating that activating the GABAergic signaling exerts an anti-inflammatory effect [17]. External GABA administration enhances gut microbiota diversity and mitigates ETEC-induced enteritis in piglets by boosting the secretion of sIgA and the anti-inflammatory cytokine IL-4 within the jejunum tissue [18]. Through the activation of GABA_A_R, GABA hinders the AMPK pathway and cellular apoptosis, thereby diminishing the harm inflicted on IPEC-J2 cells and piglet intestines caused by ETECK88 [19]. Nonetheless, contemporary research predominantly centers on GABA_A_R, while the function of GABA_B_R in the intestine remains underexplored. Prior investigations from our laboratory have revealed that activating GABA_B_R downregulates the production of inflammatory factors by impeding the TLR4/MyD88 signaling pathway, consequently easing LPS-induced intestinal inflammation in mice [20]. However, the specific mechanism of the GABA-GABA_B_R axis in the pathogenesis of intestinal disorders warrants further exploration. Based on these research backgrounds, we hypothesize that the activation of GABAergic signaling has the potential to significantly bolster antioxidant defenses, ultimately leading to amelioration of intestinal inflammatory diseases and presenting a novel therapeutic strategy for their management.

In this study, our aim is to explore the specific roles of GABAergic signaling, with a particular focus on GABA_B_R, in oxidative stress during intestinal inflammation. Our results indicate that through the activation of GABA_B_R, GABA markedly suppresses the TLR4/MyD88/NLRP3 pathway to enhance NRF2/HO-1 signaling, thereby elevating the levels of antioxidant molecules, and ultimately strengthening the antioxidant capacity in mice and IPEC-J2 cells. Our findings have the potential to uncover a new function for GABA_B_R in enteritis, offering innovative targets and crucial biological insights for treating intestinal diseases in both humans and animals.

## 2. Materials and Methods

### 2.1. Animals and Cell Lines

In the present study, we sourced adult male C57BL/6 mice, aged 6 to 8 weeks, from Beijing Vital River Laboratory Animal Technology Co., Ltd. in Beijing, China. With an average weight of 20 g, these mice were maintained in a tightly controlled environment, ensuring a consistent temperature of 20 ± 3 °C and humidity of 60 ± 5%. The mice were also subjected to a regular 12 h light/dark cycle and had unrestricted access to water and food. All experimental procedures involving these mice were reviewed and approved by the Committee for the Care and Use of Experimental Animals at China Agricultural University (AW70604202-2-1). We took substantial precautions to alleviate any potential discomfort for the animals and minimize the number of mice required for the study.

Professor Zhihui Hao’s laboratory at China Agricultural University generously provided the IPEC-J2 cells for our research. We cultivated these cells in DMEM medium, which contained 4.5 g/L of glucose and was sourced from Beijing Aoqing Biotechnology C., Ltd. in Beijing, China. This medium was enhanced with 12% heat-inactivated FBS, 2 mM glutamine obtained from Gibco in Grand Island, NY, USA, and a penicillin–streptomycin mixture supplied by BioSharp in Hefei, China. To maintain optimal conditions, the cells were kept in a humidified environment regulated at 37 °C with 5% CO_2_.

### 2.2. Development of Inflammation Models Induced by LPS Exposure

For our in vivo study, we randomly assigned 18 male mice to three separate groups: a control (CON) group, a lipopolysaccharide (LPS) group, and a group combining LPS with γ-aminobutyric acid (LPS + GABA) (6 mice per group). Mice in the LPS + GABA group received daily intraperitoneal injections of 10 mg/kg GABA (sourced from Sigma, St. Louis, MO, USA) for seven days. Mice in the CON and LPS groups, on the other hand, were given a corresponding volume of saline. After this week of pretreatment, mice were administered either an intraperitoneal injection of LPS (10 mg/kg, also from Sigma) or saline. Three hours post-injection, the mice were anesthetized using isoflurane and euthanized in a compassionate manner. We gathered ileal contents for microbiota abundance analysis and preserved ileal tissues in a 4% paraformaldehyde solution. Additionally, a portion of the ileal tissue was frozen at −80 °C for future analysis. The treatment protocol for the LPS + Baclofen group, involving the administration of Baclofen (a GABA_B_R agonist at 10 mg/kg, sourced from MCE, Monmouth Junction, NJ, USA), followed a similar approach to that of GABA, as referenced in previous studies [20,21].

In the in vitro study, we plated IPEC-J2 cells in 6-well dishes with a seeding density of 6 × 10^5^ cells per well. To simulate intestinal inflammation as it occurs in vivo, we exposed the cells to various concentrations of LPS (0.1, 1, 10, and 50 μg/mL) for 12 h. Based on this, we chose the highest concentration of 50 μg/mL LPS to challenge the cells for durations of 3, 6, and 12 h, as referenced in a previous study [22]. To evaluate the impact of GABA_B_R signaling on LPS-exposed IPEC-J2 cells, we maintained cell cultures at 37 °C in medium containing either LPS alone or LPS combined with 100 μM Baclofen, as described in previous study [23]. Control cell cultures were maintained in medium without any additives. Following the 12 h incubation, cells were collected for further analysis.

### 2.3. H&E Staining

The ileal tissue preparation for histological assessment closely followed the protocol outlined in our prior publication [24]. Briefly, the ileal tissue was fixed in 4% PFA for 48 h, dehydrated through a graded ethanol and xylene series, and paraffin embedded. The embedded samples were precisely sliced into 8 μm thick sections, optimal for staining. To identify histological changes, the ileal sections underwent hematoxylin–eosin (H&E) staining. Complete ileum cross-sections were imaged at 40× magnification using a photographic microscope (Ni-U, Nikon, Tokyo, Japan).

### 2.4. RNA Extraction and Quantitative Real-Time PCR (qRT-PCR)

Total RNA was extracted from both intestinal tissues and IPEC-J2 cells, utilizing the Ultrapure RNA Kit (CW0597S; CWBIO, Beijing, China) and strictly adhering to the manufacturer’s instructions. With the aid of the SuperRT cDNA Synthesis Kit (CW0741; CWBIO, Beijing, China), 1 μg of the extracted RNA was reverse transcribed to produce cDNA. Following the manufacturer’s guidelines, the cDNAs were combined with primers and UltraSYBR Mixture (CW0957; CWBIO, Beijing, China). The PCR amplification commenced with an initial denaturation phase at 95 °C for 10 min, succeeded by 40 cycles consisting of denaturation at 94 °C for 30 s, annealing at 60 °C for 30 s, and extension at 72 °C for 30 s. These amplifications were performed on the CFX96 Real-Time Thermal Cycler (Bio-Rad, Hercules, CA, USA). CT values were determined using the 2^−ΔΔCT^ method, normalizing with GAPDH as the reference gene. Data are presented in terms of relative fold variations compared to the CON group. For detailed primer sequences, refer to Table 1 [20].

### 2.5. Western Blotting (WB)

The total protein extracted from mouse ileum or IPEC-J2 cells underwent SDS-PAGE (E-1006; SLCY, Beijing, China) and was then transferred onto PVDF membranes (IPVH00010; Sigma, St. Louis, MO, USA). These membranes were blocked with TBST (E-1004; SLCY, Beijing, China) containing 5% nonfat milk for 1.5 h at room temperature. Following this, the membranes were exposed to various primary antibodies, such as rabbit anti-GABA_B_R (1:5000; 27567-1-AP, Proteintech, Wuhan, China), rabbit anti-NRF2 (1:2000; 16396-1-AP, Proteintech, Wuhan, China), and others including anti-HO-1, anti-iNOS, anti-TLR4, anti-MyD88, and anti-NLRP3. After incubation with the primary antibodies, the membranes were incubated with HRP-conjugated goat anti-rabbit IgG (H&L) (1:1000; A0208; Beyotime, Beijing, China) for an hour at 37 °C. The band intensities were then adjusted using mouse anti-β-actin (1:1000; 50201; Kemei Borui Science and Technology Co., Ltd., Beijing, China) as a reference. The immunoreactivity was detected by enhanced chemiluminescence (CW0049; CWBIO, Beijing, China) according to the manufacturer’s directions. The protein bands were finally imaged with a chemiluminescence system (5200; Tanon Science & Technology Co., Ltd., Shanghai, China).

### 2.6. Enzyme-Linked Immunosorbent Assay (ELISA)

To ascertain the expressions of T-AOC, SOD, and GSH, supernatants from ileal tissue samples were carefully collected and centrifuged. Subsequently, the immunoglobulins’ concentrations were precisely evaluated by utilizing an ELISA assay kit, strictly following the manufacturer’s instructions.

### 2.7. Immunofluorescence Assays

After undergoing the necessary treatment, the stabilized IPEC-J2 cells were permeabilized using 1% Triton X-100 at 37 °C for 2 h. Following this, the cells were blocked with 5% donkey serum at 37 °C for an hour and then incubated with the primary antibody, rabbit anti-GABA_B_R (1:200; 27567-1-AP, Proteintech, Wuhan, China), at 4 °C for 48 h. Subsequently, the samples were exposed to an AlexaFluor488-labeled antibody targeting rabbit IgG (1:100; A11034; Invitrogen, Carlsbad, CA, USA) for 24 h at 4 °C. The nuclei were then colored with DAPI (0100-20; SouthernBiotech, Birmingham, AL, USA) for 5 min at room temperature. The same staining procedure was applied to the negative control sections, except the primary antibodies were substituted with PBS. Imaging of the fixed IPEC-J2 cells was carried out using an Eclipse Ti-U microscope (Nikon Corp., Tokyo, Japan).

### 2.8. CCK-8 Assay

IPEC-J2 cells were seeded in 96-well plates at a density of 10,000 cells per well. Cell viability in the CON, LPS, and LPS + Baclofen groups was assessed using the CCK-8 Assay Kit (HY-K0301, MCE, Monmouth Junction, NJ, USA). Absorbance at 450 nm was measured using a microplate reader to determine cell viability.

### 2.9. Ultrahigh-Performance Liquid Chromatography–Tandem Mass Spectrometry (UHPLC-MS/MS)

Neurotransmitter levels were determined using a method established in prior studies [25,26]. The evaluation of neurotransmitter secretion in ileal tissue was entrusted to Profleader Biology Technology Co., Ltd., situated in Shanghai, China. The company utilized UHPLC-MS/MS for sample processing, calibration curve generation, data examination, and production of quantitative findings.

### 2.10. Luminex Liquid Suspension Chip Detection

The Luminex analysis was performed by Wayen Biotechnologies, situated in Shanghai, China. Utilizing the Bio-Plex Pro Mouse Cytokines Grp I Panel 23-plex kit, the expression levels of 23 cytokines in ileal tissues were quantified, adhering strictly to the manufacturer’s instructions. In brief, homogenates from ileal tissues were gathered and incubated in 96-well plates along with diluent microbeads for half an hour in a darkened setting at ambient temperature. Following the removal of supernatants, the samples underwent exposure to detection antibodies for an additional 30 min in a room temperature environment. Afterwards, Streptavidin-PE was introduced to each well and allowed to incubate for 10 min at room temperature. Ultimately, the Luminex 200 system (Luminex Corporation, Austin, TX, USA) was engaged to ascertain the expression values, as referenced in previous studies [27,28].

### 2.11. 16S rDNA Amplicon Sequencing of Ileal Microbiota

The microbiota diversity present in ileal contents was evaluated by Biomarker Technologies Co., Ltd. located in Beijing, China. They employed 16S rDNA amplicon sequencing, following a previously established protocol [21]. The recently gathered content samples were securely stored at −80 °C until required. Utilizing the SDS method, DNA was skillfully extracted, and the V3–V4 segment of 16S rDNA was successfully amplified with the help of universal primers. Subsequently, the PCR products underwent evaluation on the Illumina HiSeq platform for meticulous sequencing analysis and thorough data interpretation.

### 2.12. RNA-Sequencing

Transcriptome analysis was executed by OE Biotech Co. Ltd. situated in Shanghai, China. The retrieval of total RNA from ileal tissues was accomplished using the mirVana miRNA Isolation Kit (1561; Ambion, Austin, TX, USA), strictly adhering to the producer’s directions. The evaluation of RNA quality was aided by the Agilent 2100 Bioanalyzer (Agilent Technologies, Santa Clara, CA, USA). Sole samples boasting an RNA Integrity Number (RIN) of 7 or above were deemed eligible for further examination. The assembly of libraries was accomplished with the TruSeq Stranded mRNA LTSample Prep Kit (RS-122-2101, Illumina, San Diego, CA, USA), and these compilations were then sequenced on the Illumina sequencing framework, precisely the HiSeqTM 2500 or the Illumina HiSeq X Ten, yielding 125 bp/150 bp paired-end readings. The discovery of notably differentially expressed genes (DEGs) rested on a standard of a *p*-value under 0.05, along with a fold alteration surpassing 2 or dropping beneath 0.5.

### 2.13. Statistical Analysis

Protein gray values obtained from WB experiments were precisely quantified using Image J software Version 1.54. Data were subsequently analyzed with GraphPad Prism 9.0 software, employing *t*-tests and One-Way ANOVA to ascertain statistical significance. All experimental findings are presented as Mean ± standard error of the mean (SEM). Statistical significance, indicating notable disparities among groups, was determined by a *p*-value below 0.05. Extremely significant differences were denoted by *p*-values less than 0.01 and 0.001 [29].

## 3. Results

### 3.1. GABA Level Is Changed after LPS-Induced Intestinal Inflammation in Mice

**Figure 1:**
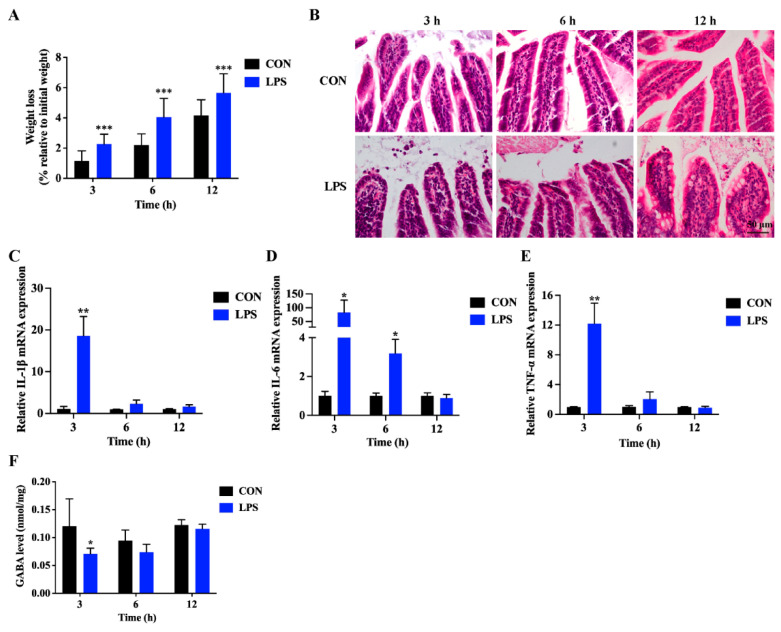
The changes of GABA level during LPS-induced intestinal inflammation. (**A**) The amount of weight loss was quantified for both the control (CON) and LPS-treated (LPS) groups at various time points. (**B**) Representative images display H&E-stained cross-sections of the ileum from both the CON and LPS groups at specific time intervals, with a 50 μm scale bar provided. (**C**–**E**) The expression levels of IL-1β, IL-6, and TNF-α mRNAs were determined through qRT-PCR analysis of mouse intestinal tissue samples collected at different times. GAPDH served as the reference gene for data normalization, and the relative fold changes in expression compared to the CON group were computed using the 2^−ΔΔCT^ method. (**F**) The levels of GABA in ileal tissue were measured using UHPLC-MS/MS at 3, 6, and 12 h post-treatment. The presented data are the mean ± SD. * indicates that the difference between the CON and the LPS is significant (*p* < 0.05). ** or *** indicates that the difference between the CON and the LPS is extremely significant (*p* < 0.01 or *p* < 0.001).

The findings showed that, in contrast to the control group (CON group), mice in the lipopolysaccharide group (LPS group) demonstrated a discernible weight reduction trend (Figure 1A). Histological examination using HE staining uncovered significant damage to the mice ileum structure caused by LPS, manifesting as mucosal hemorrhage, epithelial shedding, irregular cellular arrangement, and an infiltration of inflammatory cells (Figure 1B). qRT-PCR analysis was employed to assess the mRNA expression of inflammatory factors in the ileum tissue. The expression levels of IL-1β, IL-6, and TNF-α increased significantly at 3 h, but there was no significant change at 6 h and 12 h (Figure 1C–E). These findings imply that a 3 h intraperitoneal injection of LPS was effective in inducing intestinal inflammation in mice. Furthermore, when compared to the CON group, a substantial decrease in GABA content was observed in the ileum tissue of mice after 3 h of LPS induction, whereas no significant alterations were noted at 6 h and 12 h (Figure 1F). Interestingly, LPS also modulated the levels of other neurotransmitters, including Glu, Ach, alanine, 5-hydroxytryptamine (5-HT), methionine, serine, threonine, histamine, glutamine, and dopamine (Supplementary Figure S1). This suggests that neurotransmitters in the intestine respond rapidly to LPS-induced enteritis.

### 3.2. GABA Administration Alleviates Intestinal Damage by Improving Inflammatory Response

**Figure 2:**
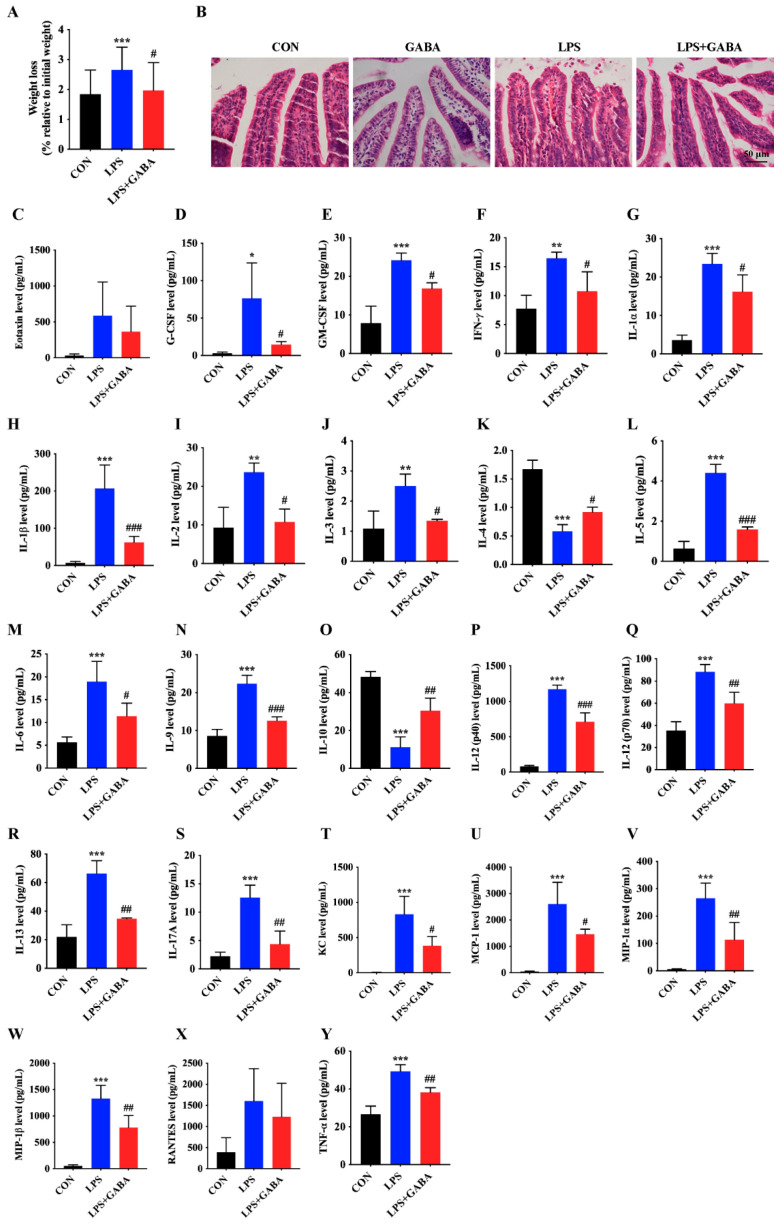
GABA attenuates intestinal mucosa injury by improving inflammatory factor expression in LPS-induced mice. (**A**) To determine weight loss, the body weights of mice were measured and compared to their initial weights at the commencement of LPS treatment. The results are presented for untreated mice (CON), mice subjected to LPS treatment (LPS), and mice treated with GABA following LPS induction (LPS + GABA). (**B**) Ileal tissues from various groups, including the control (CON), mice treated solely with GABA (GABA), mice treated with LPS, and mice treated with both LPS and GABA, were stained with H&E. The scale bar represents 50 μm. (**C**–**Y**) Inflammatory cytokines in ileal tissues were analyzed using Luminex liquid suspension chip detection. The data are presented as the mean ± SD. Statistical significance is denoted as follows: * represents a significant difference between the CON and LPS groups (*p* < 0.05), while ** or *** signifies an extremely significant difference (*p* < 0.01 or *p* < 0.001). Differences between the LPS and LPS + GABA groups are marked with # for significant (*p* < 0.05) and ## or ### for extremely significant differences (*p* < 0.01 or *p* < 0.001).

In order to examine the impact of GABA on LPS-induced intestinal inflammation in mice, a dose of 10 mg/kg of GABA was administered via intraperitoneal injection. The findings indicated that administration of GABA significantly inhibited the weight loss induced by LPS in mice (Figure 2A). HE staining revealed that GABA treatment led to well-organized ileum villi epithelia, a marked reduction in intestinal mucosal bleeding, and a notable decrease in inflammatory cell infiltration (Figure 2B). Using Luminex antibody microarray technology, we measured the concentration of inflammatory cytokines in the ileum tissue. In comparison to the CON group, the LPS group showed a significant elevation in the expression of proinflammatory cytokines, such as Eotaxin, G-CSF, GM-CSF, IFN-γ, IL-1α, IL-1β, IL-2, IL-3, IL-5, IL-6, IL-9, IL-12 (p40), IL-12 (p70), IL-13, IL-17A, KC, MCP-1, MIP-1α, MIP-1β, RANTES, and TNF-α (Figure 2C–J,L–N,P–Y). Conversely, the levels of anti-inflammatory cytokines IL-4 and IL-10 decreased notably (Figure 2K,O). Nevertheless, the administration of exogenous GABA led to a substantial decrease in proinflammatory cytokines and an increase in anti-inflammatory cytokines. These findings indicate that pretreatment with GABA could ameliorate intestinal mucosal damage caused by LPS in mice.

**Figure 3:**
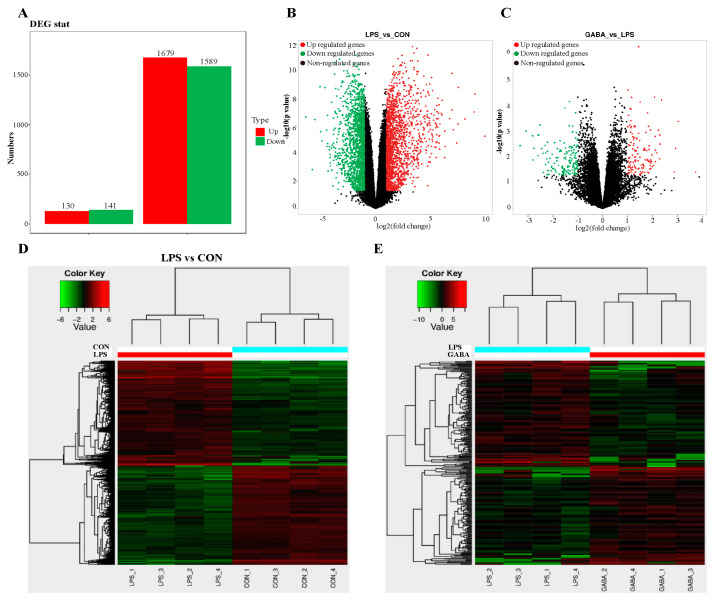

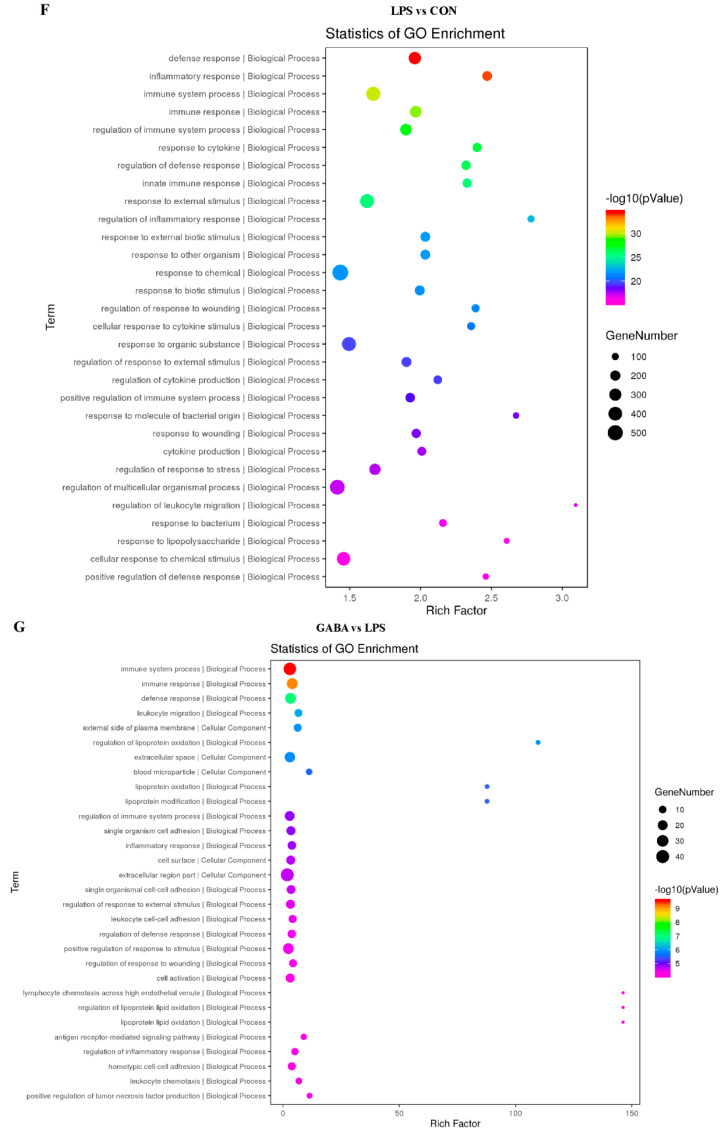

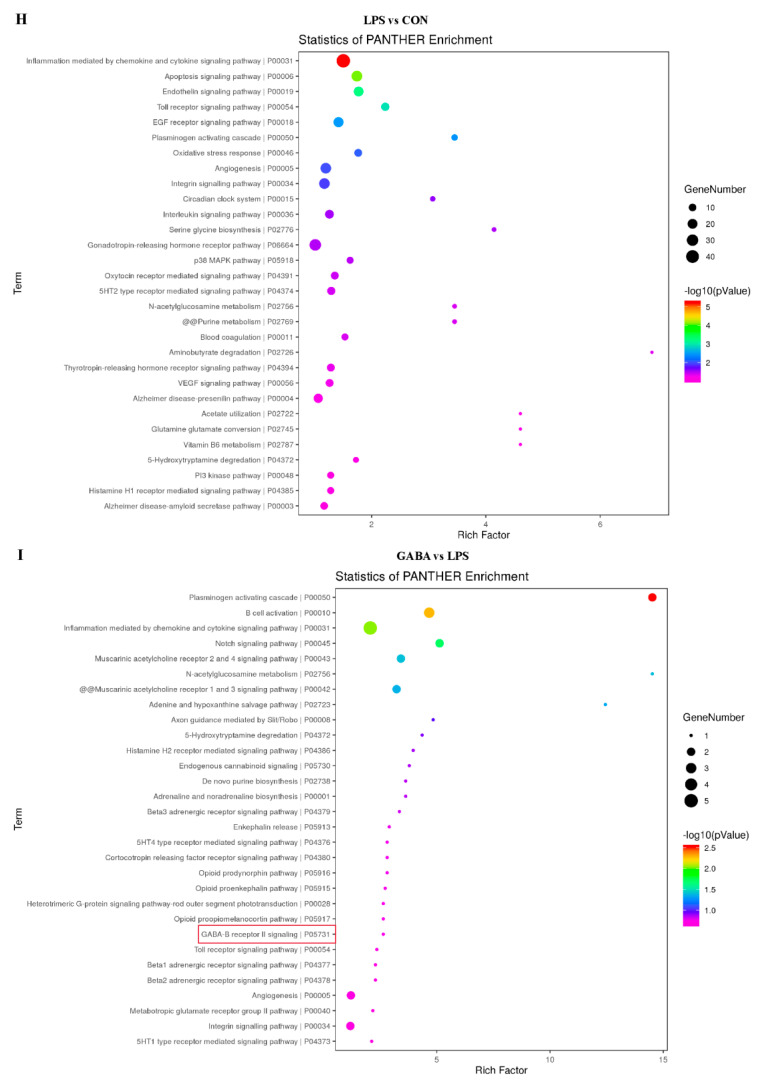
Transcriptome analysis of LPS-induced mice after GABA administration. (**A**) Total number of up- or downregulated genes of significant DEGs. (**B**,**C**) Volcano plot illustrating differentially regulated gene expression from RNA-seq analysis between LPS and CON, GABA and LPS, respectively. Genes upregulated and downregulated are shown in red and green, respectively. (**D**,**E**) Differential gene expression heat maps of mice between LPS and CON, GABA and LPS, respectively. (**F**,**G**) GO analysis showed the top 30 of enriched signaling in ileal tissues between LPS and CON, GABA and LPS, respectively. (**H**,**I**) KEGG analysis showed the top 30 of enriched signaling in ileal tissues between LPS and CON, GABA and LPS, respectively. CON: control group; LPS: LPS group; GABA: LPS + GABA treatment group.

To further confirm the underlying GABA mechanisms in mitigating intestinal damage in LPS-induced mice, we performed RNA sequencing of ileal tissues. The results showed that, in comparison to the CON group, the LPS group exhibited 3268 differentially expressed genes (Figure 3A,B). Specifically, 1679 genes were upregulated, while 1589 genes were downregulated. When compared to the LPS group, the LPS + GABA group demonstrated 271 differentially expressed genes, with 130 genes upregulated and 141 genes downregulated (Figure 3A,C). Hierarchical clustering analysis uncovered substantial disparities in gene expression patterns between the LPS and CON groups, as well as between the LPS + GABA and LPS groups (Figure 3D,E). GO enrichment and KEGG signaling pathway analyses were conducted on these differentially expressed genes. Among the top 30 most significant GO enrichment pathways, defense response, inflammatory response, and immune system processes stood out in the LPS versus CON group comparison (Figure 3F). In contrast, immune system processes, immune responses, and defense responses were more prominent in the comparison between the LPS + GABA and LPS groups (Figure 3G). An examination of the 30 most notable KEGG signaling pathways revealed that inflammation mediated by chemokine and cytokine signaling, apoptosis signaling, and endothelin signaling were more pronounced in the LPS versus CON group (Figure 3H). On the other hand, plasminogen activating cascade, B-cell activation, and inflammation mediated by chemokine, and cytokine signaling were more significant in the comparison between the LPS + GABA and LPS groups (Figure 3I). Furthermore, our findings indicated that exogenous GABA administration significantly enriched GABA_B_R signaling (Figure 3I, as denoted by the red box). Collectively, these results suggest that through activation of GABA_B_R, GABA could regulate LPS-induced intestinal inflammation at the gene expression level.

### 3.3. Activation of GABA_B_R Modulates Intestinal Inflammation by Modulating Inflammatory Factors and Oxidative Stress In Vivo and In Vitro

**Figure 4:**
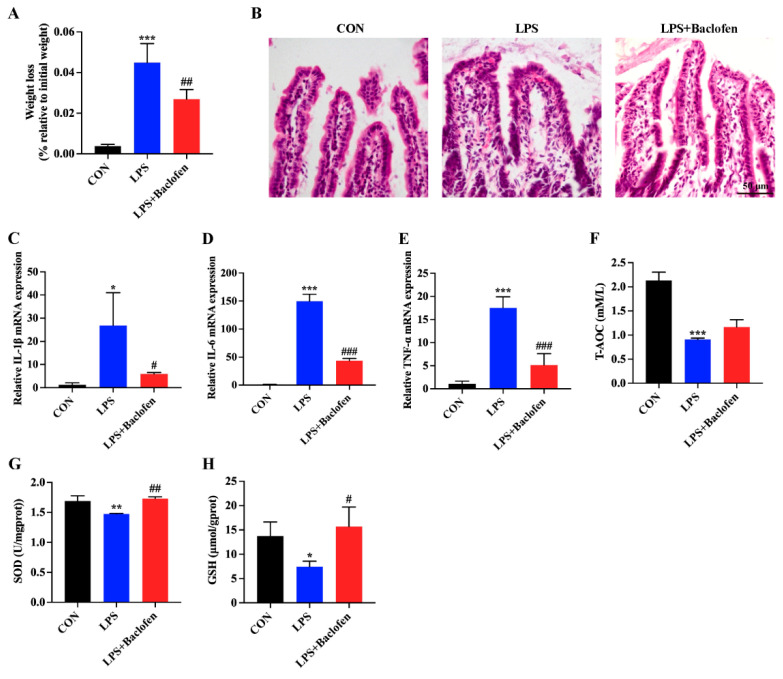
GABA_B_R activation could reduce the levels of inflammatory cytokine and oxidative stress in LPS-induced mice. (**A**) To assess weight loss, the body weights of mice were measured and compared to their weights recorded at the start of LPS treatment. The presented results cover untreated mice (CON), mice treated with LPS, and mice treated with Baclofen following LPS induction (LPS + Baclofen). (**B**) Ileal tissues from the CON, LPS, and LPS + Baclofen mice groups underwent H&E staining. The scale bar represents 50 μm. (**C**–**E**) qRT-PCR was employed to determine the mRNA levels of IL-1β, IL-6, and TNF-α, with GAPDH serving as the reference gene. The average fold changes compared to the CON group were calculated using the 2^−ΔΔCT^ method. (**F**–**H**) The graphs depict the expression levels of T-AOC, SOD, and GSH in ileal tissues. Data are presented as mean ± SEM. Statistical significance is denoted by asterisks: * for significant differences between CON and LPS (*p* < 0.05), ** or *** for extremely significant differences (*p* < 0.01 or *p* < 0.001). # indicate significant differences between the LPS and LPS + Baclofen groups, with # for significant (*p* < 0.05) and ## or ### for extremely significant differences (*p* < 0.01 or *p* < 0.001).

The aforementioned experimental outcomes imply that GABA potentially exerts its effect via the GABA_B_R signaling. Moreover, prior investigations conducted in our laboratory have revealed a notable decrease in GABA_B_R expression during intestinal inflammation [20], prompting us to primarily focus on the GABA_B_R signaling. To delve into the effects of GABA_B_R activation on LPS-induced mice, we administered Baclofen (GABA_B_R agonist) intraperitoneally to mice for five consecutive days. The results showed that LPS stimulation led to a considerable reduction in the body weight of the mice, whereas the introduction of Baclofen mitigated this weight loss (Figure 4A). Upon LPS exposure, the ileal villi of the mice exhibited evident damage, including disrupted intestinal epithelial cell arrangement, substantial lymphocyte infiltration, and hemorrhaging (Figure 4B). In contrast, mice in the LPS + Baclofen group exhibited lesser ileal tissue damage, with more orderly arranged intestinal epithelial cells and minimal lymphocyte infiltration and hemorrhaging, suggesting that activating GABA_B_R could ameliorate ileal tissue damage inflicted by LPS. Utilizing qRT-PCR, we assessed the mRNA levels of inflammatory cytokines in the ileal tissue. Mice in the LPS group demonstrated elevated expression of the proinflammatory cytokines IL-1β, IL-6, and TNF-α compared to the CON group (Figure 4C–E). Notably, administration of Baclofen significantly reversed this aberrant inflammatory cytokine expression. Oxidative stress constitutes a pivotal aspect of intestinal inflammatory disease [30]. Consequently, we evaluated the oxidative stress indicators in each group. Compared to the CON group, the LPS group exhibited obviously diminished levels of T-AOC, SOD, and GSH, suggesting that the antioxidant capacity was weakened in mice (Figure 4F–H). Remarkably, Baclofen administration notably augmented the expression of these molecules, thereby bolstering the antioxidant capabilities of mice to some extent. In conclusion, our findings underscore that the activation of GABA_B_R mitigates LPS-induced intestinal inflammation in mice by suppressing proinflammatory cytokine levels and enhancing antioxidant capacity.

**Figure 5:**
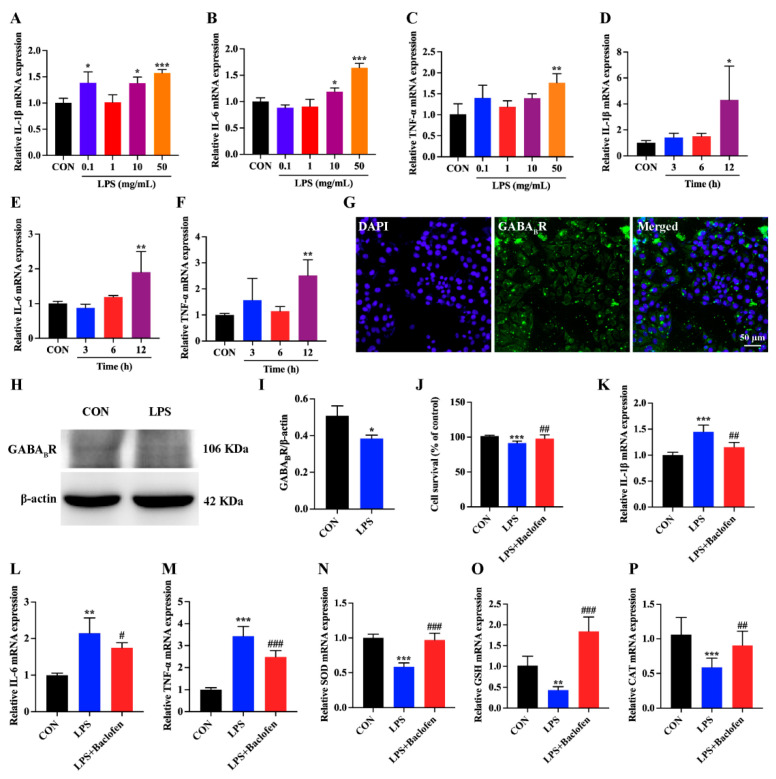
GABA_B_R activation could reduce the levels of inflammatory cytokine and oxidative stress in IPEC-J2 cells. (**A**–**C**) qRT-PCR was utilized to determine the mRNA levels of IL-1β, IL-6, and TNF-α in IPEC-J2 cells treated with varying concentrations of LPS (0.1, 1, 10, 50 mg/mL). (**D**–**F**) The mRNA expression of IL-1β, IL-6, and TNF-α was assessed by qRT-PCR at different time intervals following LPS administration to IPEC-J2 cells. (**G**) Cultured IPEC-J2 cells were subjected to single immunofluorescence staining for GABA_B_R. The scale bar represents 50 μm. (**H**,**I**) Western blot (WB) analysis was conducted to examine the expression of GABA_B_R in IPEC-J2 cells. (**J**) Cell viability assessments were performed on IPEC-J2 cells in the CON, LPS, and LPS + Baclofen groups. (**K**–**M**) The mRNA levels of IL-1β, IL-6, and TNF-α were measured by qRT-PCR in the CON, LPS, and LPS + Baclofen groups. (**N**–**P**) The levels of SOD, GSH, and CAT were determined in the same groups. For qRT-PCR, GAPDH was used as the reference gene, and fold changes relative to the CON group were calculated using the 2^−ΔΔCT^ method. For WB, protein expression was normalized against β-actin. Data are presented as mean ± SEM. Statistical significance is denoted by asterisks: * for significant differences between CON and LPS (*p* < 0.05), ** or *** for extremely significant differences (*p* < 0.01 or *p* < 0.001). # indicate significant differences between the LPS and LPS + Baclofen groups, with # for significant (*p* < 0.05) and ## or ### for extremely significant differences (*p* < 0.01 or *p* < 0.001).

Intestinal epithelial cells constitute a vital protective barrier, safeguarding the body from invading pathogenic microorganisms and toxins [31]. The Toll-like receptors on their surface are adept at recognizing LPS, which stimulates an augmented production of proinflammatory cytokines and disrupts the oxidative stress balance, with the potential to cause cellular harm [32]. We crafted an LPS concentration gradient, including 0.1, 1, 10, and 50 mg/mL, and assessed inflammatory factor expression in IPEC-J2 cells using qRT-PCR. Compared to the CON group, we noticed a marked surge in IL-1β mRNA expression at LPS concentrations of 0.1, 10, and 50 mg/mL (Figure 5A). Analogously, IL-6 mRNA expression significantly rose at 10 and 50 mg/mL, while TNF-α mRNA expression saw a notable increase only at 50 mg/mL (Figure 5B,C). As the LPS exposure time prolonged, the expression levels of IL-1β, IL-6, and TNF-α mRNA also escalated in a time-dependent manner versus the CON group (Figure 5D–F). Hence, we determined that an LPS concentration of 50 mg/mL, with a 12 h induction period, was most suitable for our subsequent investigations. To explore GABA_B_R expression in IPEC-J2 cells, we resorted to immunofluorescence methods. Our observations indicated substantial GABA_B_R expression in untreated IPEC-J2 cells (Figure 5G). Nevertheless, GABA_B_R protein expression significantly diminished upon LPS treatment, signifying GABA_B_R signaling suppression (Figure 5H,I). We further probed the effect of GABA_B_R activation on LPS-induced IPEC-J2 cells. The results indicated that LPS notably decreased the viability of IPEC-J2 cells (Figure 5J). However, Baclofen pretreatment markedly counteracted this detrimental trend, elevating it to the level observed in the CON group, suggesting that activating GABA_B_R could improve the survival rate of IPEC-J2 cells exposed to LPS. Our findings showed that, relative to the CON group, IL-1β, IL-6, and TNF-α mRNA expression increased notably in the LPS group (Figure 5K–M). Remarkably, baclofen pretreatment led to a considerable diminution in these proinflammatory factors, suggesting that activating GABA_B_R in IPEC-J2 cells exerts anti-inflammatory actions. We next used qRT-PCR to assess the mRNA level of antioxidant molecules. Our results pointed to a notable decline in SOD, GSH, and CAT expression after LPS treatment, indicating reduced antioxidant capacity in IPEC-J2 cells (Figure 5N–P). Intriguingly, Baclofen pretreatment significantly increased the expression of these molecules. The above data suggest that GABA_B_R activation could enhance the antioxidant capability of LPS-induced IPEC-J2 cells.

### 3.4. Activation of GABA_B_R Reduce Oxidative Stress through Inhibiting TLR4/MyD88/NLRP3 Pathway

**Figure 6:**
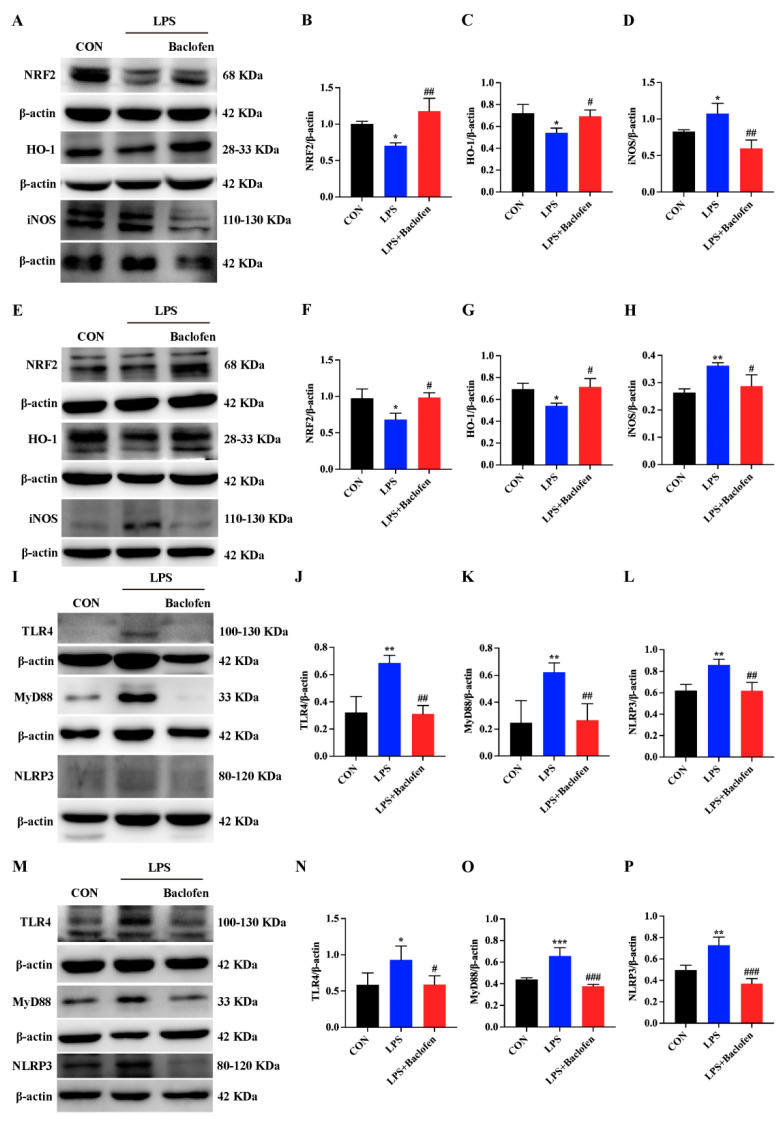
GABA_B_R activation could ameliorate oxidative stress by inhibiting TLR4/MyD88/NLRP3 in vivo and in vitro. (**A**–**D**) WB analysis of the expression of NRF2, HO-1, and iNOS in ileal tissues of mice. (**E**–**H**) WB analysis of the expression of NRF2, HO-1, and iNOS in IPEC-J2 cells. (**I**–**L**) WB analysis of the expression of TLR4, MyD88, and NLRP3 in ileal tissues of mice. (**M**–**P**) WB analysis of the expression of TLR4, MyD88, and NLRP3 in IPEC-J2 cells. For WB, protein expression was normalized against β-actin. Data are presented as mean ± SEM. Statistical significance is denoted by asterisks: * for significant differences between CON and LPS (*p* < 0.05), ** or *** for extremely significant differences (*p* < 0.01 or *p* < 0.001). # indicate significant differences between the LPS and LPS + Baclofen groups, with # for significant (*p* < 0.05) and ## or ### for extremely significant differences (*p* < 0.01 or *p* < 0.001).

To delve into the regulatory mechanisms of GABA_B_R in oxidative stress triggered by LPS in mice and IPEC-J2 cells, we employed WB to assess the expression of proteins linked to the oxidative stress pathway. Our findings revealed that LPS administration caused a downregulation of NRF2 and HO-1 levels in both mice intestine and IPEC-J2 cells, coupled with an elevation in iNOS expression (Figure 6A–H). Remarkably, pretreatment with Baclofen effectively reversed these molecular irregularities. Furthermore, our exploration also uncovered that NRF2 and HO-1 expressions were suppressed in the jejunum and ileum tissues of mice infected with ETECK88, a condition that was significantly mitigated by pretreatment with Baclofen (Supplementary Figure S2). Previous research has established that suppressing the TLR4/MyD88 signaling pathway elevates cellular antioxidant capacity [33]. Through WB analysis, we observed that LPS significantly induced high expression of TLR4, MyD88, and NLRP3 proteins in mice intestine and IPEC-J2 cells (Figure 6I–P). Nevertheless, Baclofen pretreatment showed a substantial reversal of this trend. These observations strongly suggest that activation of GABA_B_R boosts the antioxidant capacity of LPS-exposed mice and IPEC-J2 cells by impeding the TLR4/MyD88 pathway to elevate NRF2 and HO-1 expression.

### 3.5. GABA_B_R Modulates the Gut Microbiota in LPS-Induced Mice

**Figure 7:**
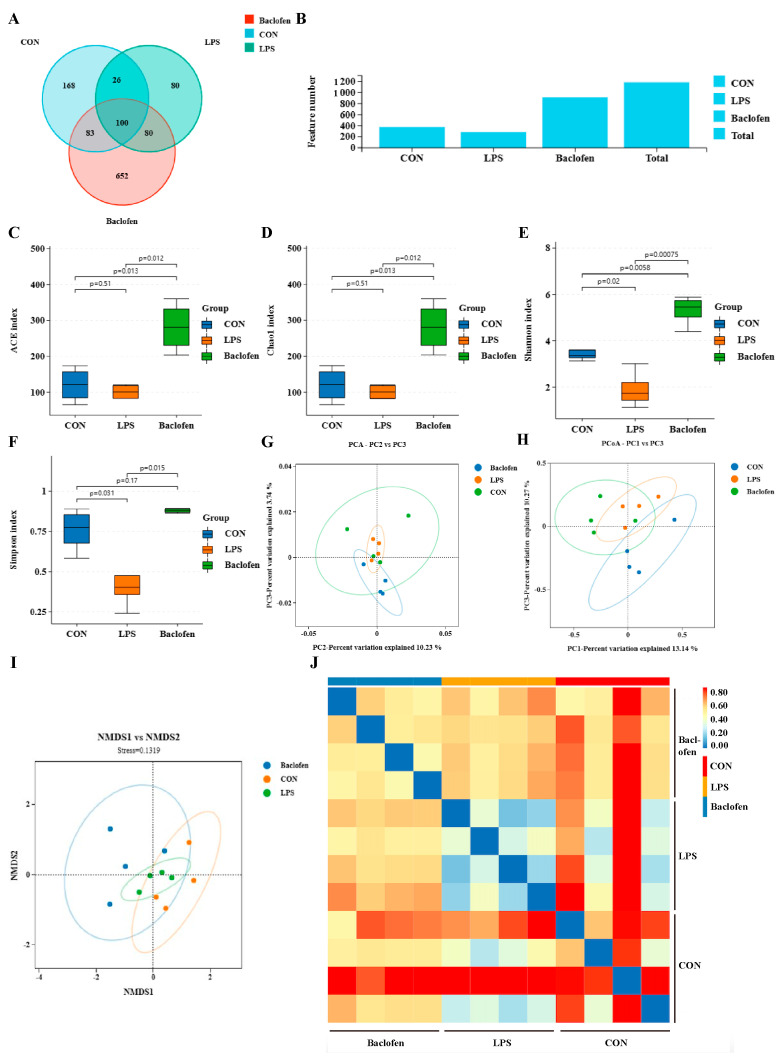
Diversity indexes of microbiota in the mice intestine. (**A**) Venn diagram of the number of OTUs. (**B**) OTU number histogram. (**C**–**F**) α-diversity analyzed by ACE, Chao1, Shannon and Simpson indices. (**G**–**J**) Principal component, principal coordinates, NMDS and sample clustering heat map analyses of gut microbiota of each group. CON: control group; LPS: LPS group; Baclofen: LPS + Baclofen treatment group.

Maintaining the balance of gut microbiota, which holds profound importance in alleviating oxidative stress, is essential for warding off intestinal diseases caused by oxidative damage [34,35,36]. To further understand the effects of GABA_B_R activation on microbiota in the gut, we performed 16S rDNA sequencing. The results showed that the CON group, LPS group, and LPS + Baclofen group had OTU counts of 168, 80, and 652, respectively (Figure 7A). Notably, there were 100 OTUs common to all three groups. In comparison to the CON group, the LPS group exhibited a substantial decrease in OTU count, whereas the LPS + Baclofen group showed a marked increase, suggesting that Baclofen therapy significantly enhances the diversity of intestinal microbiota (Figure 7B). α-diversity offers a metric for assessing species richness and diversity within a sample, often quantified by the ACE, Chao1, Shannon, and Simpson indices. In contrast to the CON group, the LPS group showed marked reductions in these indices, indicating a significant disturbance to the intestinal species diversity in mice (Figure 7C–F). Nevertheless, Baclofen pretreatment had the potential to counteract this abnormal alteration. More interestingly, PCA, PCoA, NMDS, and sample clustering heat map analyses uncovered remarkable disparities in the traits of the ileal flora communities among the various groups of mice (Figure 7G–J). Furthermore, the intestinal flora of mice pretreated with Baclofen for acute enteritis bore resemblance to that of healthy mice.

**Figure 8:**
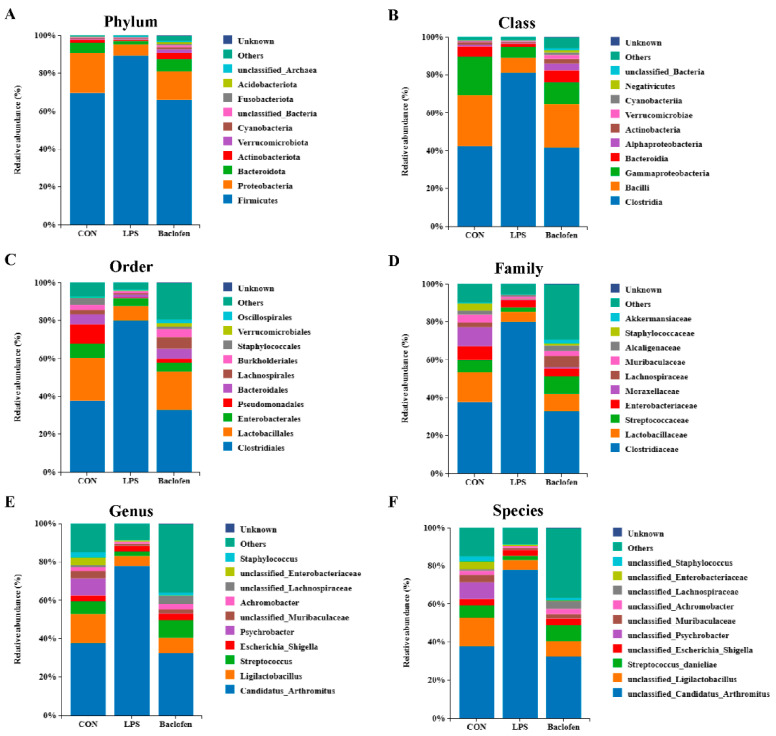

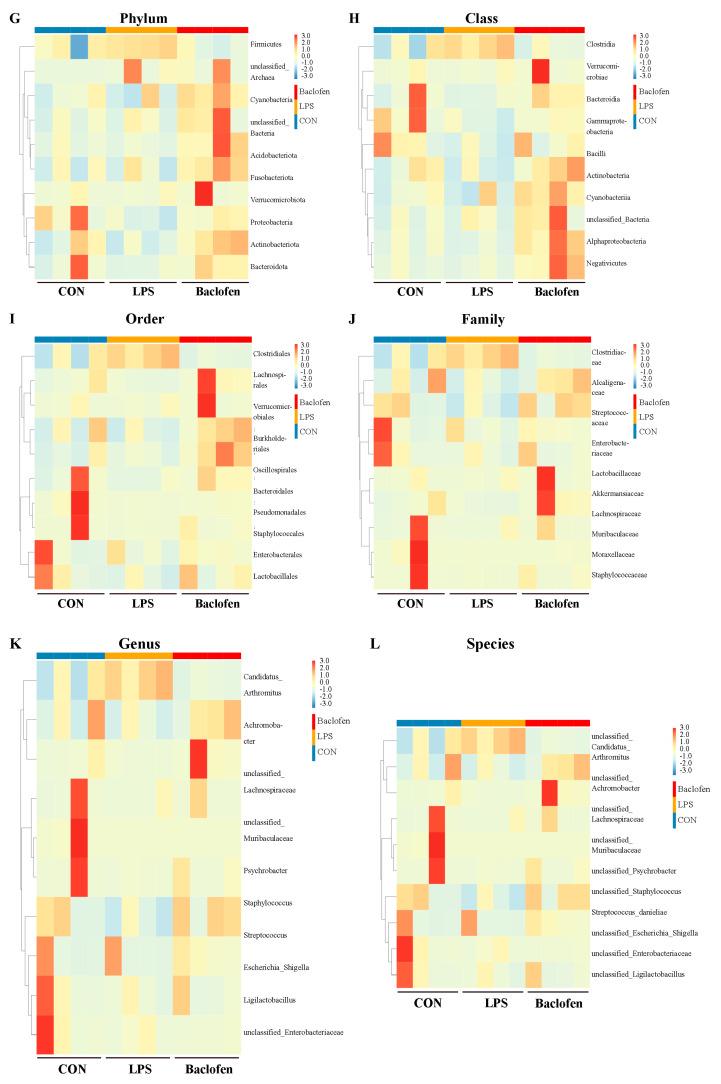
Analysis of the microbial composition. (**A**–**F**) 16S rDNA sequencing results of relative abundance of intestinal bacteria in mice at phylum, class, order, family, genus, and species levels in each group. (**G**–**L**) Cluster heatmap of species abundance in each group. CON: control group; LPS: LPS group; Baclofen: LPS + Baclofen treatment group.

We further explored the disparities in the relative abundance of ileal microbiota across various treatment groups, scrutinizing them at the levels of phylum, class, order, family, genus, and species. In comparison to the CON group, the LPS group exhibited an increase in the relative abundance of *Firmicutes* at the phylum level, while *Proteobacteria*, *Bacteroidota*, and *Actinobacteriota* decreased (Figure 8A,G). At the class level, *Clostridia* emerged as the microbiota with heightened relative abundance, whereas *Bacilli*, *Gammaproteobacteria*, and *Bacteroidia* saw a reduction (Figure 8B,H). Moving to the order level, *Clostridiales* dominated, whereas *Lactobacillales*, *Enterobacterales*, *Pseudomonadales*, *Bacteroidales*, *Lachnospirales*, and *Burkholderiales* diminished (Figure 8C,I). At the family level, *Clostridiaceae* flourished, contrasting with the decline in *Lactobacillaceae*, *Streptococcaceae*, *Enterobacteriaceae*, *Moraxellaceae*, *Lachnospiraceae*, and *Muribaculaceae* (Figure 8D,J). On the genus level, *Candidatus_Arthromitus* and *Escherichia_Shigella* thrived, while *Ligilactobacillus*, *Streptococcus*, *Pseudomonas*, *unclassified_Muribaculaceae*, and *Achromobacter* dwindled (Figure 8E,K). Finally, at the species level, *unclassified Candidatus_Arthromitus* and *unclassified Escherichia_Shigella* gained ground, whereas *unclassified_Ligilactobacillus*, *Streptococcus_danieliae*, *unclassified_Psychrobacter*, *unclassified_Muribaculaceae*, *unclassified_Achromobacter*, *unclassified_Enterobacteriaceae*, and *unclassified_Staphylococcus* lost footing (Figure 8F,L). Nevertheless, Baclofen intervention notably reversed the microbiota imbalances induced by LPS in mice, predominantly by bolstering the relative abundance of beneficial bacteria, in particular *Lactobacillus*, a bacterium possessing powerful antioxidant properties (Figure 8A–L). These data suggest that activation of GABA_B_R could modulate the relative richness of gut microbiota, ultimately decreasing oxidative stress in mice challenged with LPS.

## 4. Discussion

Our findings demonstrate that through activation of GABA_B_R, GABA potently inhibits the TLR4/MyD88/NLRP3 pathway, leading to the enhancement of NRF2/HO-1 signaling. This cascade of events suppresses the expression of proinflammatory cytokines, elevates the levels of antioxidant molecules, and consequently fortifies the antioxidant capacity in both mice and IPEC-J2 cells. Moreover, activation of GABA_B_R by Baclofen significantly augments the relative abundance of probiotics, notably increasing *Lactobacillus*, which is related to antioxidant activity, and ultimately reducing LPS-induced oxidative stress damage in mice.

GABA, a pivotal inhibitory neurotransmitter in the central nervous system, also plays a significant role in the gastrointestinal tract. The chief sources of GABA in the intestine are the highly active GABAergic neurons situated within the myenteric plexus, and mucosal endocrine-like cells, emphasizing its dual role as a neuromediator and endocrine mediator [37]. Exogenous GABA supplementation significantly reduces ETEC-induced intestinal inflammation in piglets by stimulating increased secretion of sIgA in the jejunum, amplifying the expression of IL-4, IL-13, and IL-17, and bolstering the abundance of *Enterococcus* and *Bacteroidetes* [18]. Furthermore, intestinal microbiota-derived GABA ameliorates mice intestinal inflammation provoked by ETEC or Citrobacter infection, mediated through the activation of the mTORC1-S6K1 signaling pathway to elevate IL-17 levels in intestinal tissue [38]. Our results clearly indicate that GABA pretreatment significantly attenuated LPS-induced intestinal damage in mice. This amelioration was primarily due to decreased weight loss, reduced inflammatory cell infiltration in the intestinal mucosa, and minimized epithelial cell shedding. Moreover, GABA downregulated the intestinal tissue expression of numerous proinflammatory cytokines in mice, such as Eotaxin, G-CSF, GM-CSF, IFN-γ, IL-1α, IL-1β, IL-2, IL-3, IL-5, IL-6, IL-9, IL-12 (p40), IL-12 (p70), IL-13, IL-17A, KC, MCP-1, MIP-1α, MIP-1β, RANTES, and TNF-α. Simultaneously, it enhanced the levels of anti-inflammatory cytokines IL-4 and IL-10. Given that these inflammatory mediators are mainly secreted by immune cells during inflammatory processes [39,40], it indicates that GABA may possess immune-regulatory properties. We also observed that stimulating mice with LPS caused significant enrichment in defense, inflammatory, and immune responses, as evidenced by GO analysis. Simultaneously, KEGG analysis emphasized the inflammatory signaling pathways mediated by chemokines and cytokines, and Toll-like receptor signaling pathways. These pathways are intimately linked to inflammation, further highlighting the effectiveness of LPS in inducing intestinal inflammation in mice. However, pretreatment with GABA shifted the focus of the GO analysis towards immune system processes. Likewise, KEGG analysis identified enrichment in B-cell activation, suggesting that GABA possesses the ability to regulate the immune system during inflammatory responses. Moreover, our study demonstrated a substantial enrichment of the GABA_B_R signaling by GABA, indicating a potential critical role for the GABA-GABA_B_R axis in alleviating intestinal inflammation.

To date, the specific role of GABA_B_R in intestinal inflammation remains sparse. It has been reported that activating GABA_B_R with Baclofen could alleviate intestinal pain symptoms, primarily via suppressing the threshold reaction caused by colonic dilation in rats [37]. Furthermore, GABA_B_R serves as a vital intermediary, enabling the colon to sense external stimuli and consequently mitigate associated pathological impairments [37]. However, existing studies are insufficient to comprehensively elucidate the exact action played by GABA_B_R in intestinal function. Our study revealed that administering an intraperitoneal injection of 10 mg/kg Baclofen five days before LPS induction notably mitigated intestinal damage in mice. This positive outcome was predominantly reflected in the diminution of diarrhea symptoms, an improvement in mental state, a decrease in weight loss rate and spleen index, reduced intestinal mucosal hemorrhage and congestion, preserved intestinal epithelial cell structure, and diminished lymphocyte infiltration. Most notably, Baclofen effectively suppressed the levels of proinflammatory cytokines such as IL-1β, IL-6, and TNF-α mRNA, highlighting the potent anti-inflammatory impact of GABA_B_R activation. Given that cytokines during inflammatory responses predominantly originate from immune cells, our findings implicated a potential role of GABA_B_R in modulating the immune system. Nonetheless, current research indicates that T cells, dendritic cells, macrophages, and microglia predominantly express GABA_A_R [15,41]. Whether these immune cells also express GABA_B_R remains an area of further investigation. In addition, oxidative stress damage may result in inflammation, frequently accompanied by a disruption in the balance between oxidation and antioxidation systems [42,43]. Recently, emerging research has indicated that GABA possesses the ability to regulate crucial inflammatory responses and immune cell activities, showcasing its antioxidant and anti-inflammatory capabilities. In a study utilizing a rat model with Letrozole-induced polycystic ovary syndrome, GABA treatment demonstrated a protective effect by enhancing antioxidant defense mechanisms and reducing insulin resistance [44]. Throughout various investigations, it has been consistently documented that GABA offers significant shielding from H_2_O_2_-induced oxidative stress in both pancreatic and human umbilical vein endothelial cells, with its safeguarding effect becoming apparent through decreased cell death rates, suppressed production of reactive oxygen species (ROS), and fortified antioxidant defense mechanisms [45]. Our data demonstrated that during LPS-induced enteritis in mice, there was a significant decrease in the expression of antioxidant molecules such as T-AOC, SOD, and GSH, implying a reduction in the mice antioxidant capabilities. Remarkably, Baclofen significantly elevated the expression of these antioxidant molecules, indicating that activating GABA_B_R could boost the antioxidant defenses of mice.

Intestinal epithelial cells are pivotal in defending the body against invading pathogenic microorganisms and toxins, thus preserving the delicate equilibrium of the intestinal mucosal barrier [46]. However, oxidative stress represents a serious hazard, capable of causing harmful effects to intestinal epithelial cells and potentially resulting in the collapse of the intestinal mucosal barrier [47,48]. Research reveals that LPS, a pivotal constituent of the cell wall in Gram-negative bacteria, possesses the unique capability to identify Toll-like receptors situated on the surface of intestinal epithelial cells [49,50]. This identification triggers the activation of the NF-κB signaling pathway, prompts the production of chemokines and inflammatory cytokines, and sets off a chain of oxidative stress reactions. Therefore, we moved forward to explore the impact of GABA_B_R signaling on IPEC-J2 cells stimulated by LPS. To begin, we verified the expression of GABA_B_R in IPEC-J2 cells, and our results indicated its notable presence in these cells. Unfortunately, exposure to LPS significantly reduced GABA_B_R levels, suggesting the inhibition of GABA_B_R signaling. Additionally, stimulating IPEC-J2 cells with LPS resulted in a considerable rise in the levels of proinflammatory cytokines, specifically IL-1β, IL-6, and TNF-α, while simultaneously lowering the expression of SOD, GSH, and CAT. This suggests that LPS strongly provoked inflammatory reactions in IPEC-J2 cells and undermined their antioxidant defenses. Delightedly, pretreatment with Baclofen greatly reduced the secretion of the proinflammatory cytokines IL-1β, IL-6, and TNF-α, indicating that activating GABA_B_R in IPEC-J2 cells exerts an anti-inflammatory function. This revelation is consistent with our in vivo experiments. Moreover, we observed that activating GABA_B_R notably boosted SOD, GSH, and CAT expression. To some extent, our findings suggest that GABA_B_R activation exhibits antioxidant properties in IPEC-J2 cells.

NRF2 is a highly recognized cytoprotective transcription factor that plays a vital role in defending cells against tissue damage resulting from diverse oxidative stresses [51]. When oxidative stress is encountered by cells, NRF2 migrates to the cell nucleus and upregulates the production of antioxidant elements, including HO-1 and NQO1, effectively shielding cells from damage [51,52]. HO-1, a key NRF2 target gene, is instrumental in maintaining redox balance and mitigating inflammatory responses [52]. It aids in the breakdown of heme into bilirubin, a potent natural antioxidant, counteracting oxidative insults. Studies have shown that elevated levels of HO-1 reinforce the protective mechanisms of cells during oxidative challenges [51]. Our data indicated that LPS obviously lowered NRF2 and HO-1 expression in mice and IPEC-J2 cells, while simultaneously increasing iNOS levels, suggesting a weakness in the antioxidant system. We then explored whether activating GABA_B_R could strengthen the antioxidant defenses. Impressively, pretreatment with Baclofen led to notable enlargement in NRF2 and HO-1 levels, while impairing iNOS expression, suggesting that activating GABA_B_R in mice and IPEC-J2 cells enhances the antioxidant capabilities. The TLR4/MyD88 pathway is a well-known inflammatory pathway that becomes activated during inflammatory processes, leading to incremental production of proinflammatory cytokines [53]. Our study showed that Baclofen pretreatment effectively depressed the high expression of TLR4, MyD88, and NLRP3 proteins induced by LPS in both mice and IPEC-J2 cells, indicating suppression of the TLR4/MyD88/NLRP3 signaling cascade through GABA_B_R activation. Previous studies have evidenced that inhibiting the TLR4/MyD88 pathway dramatically restrains oxidative stress-induced damage in mice [32,54]. Based on these findings, we postulated that activating GABA_B_R might intensify NRF2/HO-1 signaling by weakening the TLR4/MyD88/NLRP3 pathway, thereby heightening the antioxidant defenses of mice and IPEC-J2 cells.

The gut microbiota is crucial in the initiation and development of several diseases, such as enteritis, obesity, cancer, and cirrhosis [55,56]. Specific bacteria within the gut microbiota possess the capability to generate bodily beneficial compounds through their metabolism, influencing redox reactions and consequently easing cellular injury induced by oxidative stress [9,57,58]. However, during intestinal inflammation, the diversity of microbiota can be affected. By employing 16S rDNA sequencing, we observed an apparent reduction in species diversity of gut microbiota in mice after LPS induction, indicating a substantial disturbance to the microbiota. Interestingly, pretreatment with Baclofen significantly improved the gut microbiota profile in LPS-induced mice, aligning it closely with that of the healthy control mice. This suggests that activating GABA_B_R could aid in maintaining the balance of gut microbiota. We further explored the variations in the relative abundance of microbiota among different treatment groups at multiple taxonomic levels, including phylum, order, class, family, genus, and species. In contrast to the CON group, the LPS group showed an increase in the relative abundance of *Firmicutes*, *Clostridia*, *Clostridiales*, *Clostridiaceae*, *Candidatus_Arthromitus*, and *unclassified Candidatus_Arthromitus*. Conversely, the relative abundance of *Proteobacteria*, *Bacilli*, *Lactobacillale*, *Lactobacillaceae*, *Ligilactobacillus*, and *unclassified_Ligilactobacillus* decreased, suggesting a rise in harmful bacteria. However, the administration of Baclofen significantly reversed the microbiota imbalance caused by LPS in mice, primarily by increasing the relative abundance of beneficial bacteria, particularly *Lactobacillus*. These beneficial bacteria release acidic compounds like lactic acid and hydrogen peroxide, which lower the pH of the surrounding environment and hinder the growth of harmful bacteria that thrive in alkaline conditions. As a result, this preserves the equilibrium of gut microbiota. Additionally, due to their outstanding capacity to stimulate the immune system, these probiotics effectively bolster both the specific and nonspecific immune responses within the body. More importantly, there is numerous research indicating that *Lactobacillus*, whose multiple metabolic pathways are closely tied to antioxidant reactions, notably heightens the antioxidant defenses of the host [59,60,61]. This strengthening occurs primarily through the synthesis of antioxidant substances, such as hydrogen peroxide, which in turn stimulates the antioxidant enzyme system. *Lactobacillus* also intensifies the activity of specific antioxidant enzymes, among which SOD, GSH, and GPx are included, to efficiently neutralize harmful free radicals. Therefore, based on our results, we believed that the activation of GABA_B_R reduced intestinal damage in mice caused by oxidative stress, and this beneficial effect could, to some extent, be linked to the higher abundance of *Lactobacillus*.

## 5. Conclusions

In summary, our study demonstrates that activation of GABA_B_R enhances the expression of NRF2 and HO-1 by blocking the TLR4/MyD88/NLRP3 pathway, resulting in elevated levels of antioxidant molecules including T-AOC, SOD, GSH, and CAT, thus reinforcing the antioxidant defenses of mice and IPEC-J2 cells. Moreover, GABA_B_R activation enriches gut microbiota diversity by increasing beneficial bacteria, especially *Lactobacillus*, which has potent antioxidant properties, and ultimately eases oxidative stress to alleviate LPS-induced intestinal inflammation in mice.

## Figures and Tables

**Table 1:** Specific primers used in qRT-PCR analysis.

Gene	Direction	Primer Sequence (5′→3′)
*Mouse IL-1β*	Forward Reverse	GCTGCTTCCAAACCTTTGAC AGCTTCTCCACAGCCACAAT
*Mouse IL-6*	Forward Reverse	TAGTCCTTCCTACCCCAATTTCC TTGGTCCTTAGCCACTCCTTC
*Mouse TNF-α*	Forward Reverse	CCGATGGGTTGTACCTTGTC AGATAGCAAATCGGCTGACG
*Mouse GAPDH*	Forward Reverse	TTCCTACCCCCAATGTATCCG CATGAGGTCCACCACCCTGTT
*Pig IL-1β*	Forward Reverse	TCAGCACCTCTCAAGCAGAA GACCCTCTGGGTATGGCTTT
*Pig IL-6*	Forward Reverse	TTCACCTCTCCGGACAAAAC TCTGCCAGTACCTCCTTGCT
*Pig TNF-α*	Forward Reverse	TTCCAGCTGGCCCCTTGAGC GAGGGCATTGGCATACCCAC
*Pig SOD*	Forward Reverse	CAGGGCACCATCTACTTCGAG CAACGTGCCTCTCTTGATCCT
*Pig GSH*	Forward Reverse	CCTCAAGTACGTCCGACCAG GTGAGCATTTGCGCCATTCA
*Pig CAT*	Forward Reverse	CGAAGGCGAAGGTGTTTG CAAACCCACGAGGGTCAC
*Pig GAPDH*	Forward Reverse	TGTCCACCTTCCAGCAGATGT AGCTCAGTAACAGTCCGCCTAGA

## Data Availability

All data that support the findings of this study are available from the corresponding author upon reasonable request.

## Supplementary Materials

The following supporting information can be downloaded at: https://www.mdpi.com/article/10.3390/antiox13091141/s1, Figure S1: Neurotransmitter levels in ileal tissue at different time points; Figure S2: GABA_B_R activation could reduce oxidative stress in ETECK88-induced mice.
