# Supplementary material for: Activation of GABABR Attenuates Intestinal Inflammation by Reducing Oxidative Stress through Modulating the TLR4/MyD88/NLRP3 Pathway and Gut Microbiota Abundance
Source: Antioxidants (Basel). 2024 Sep 21;13(9):1141. doi: 10.3390/antiox13091141 (PMC11428452; doi:10.3390/antiox13091141)
Supplement: Supplementary file 1 [file antioxidants-13-01141-s001.zip › antioxidants-3075311-supplementary.pdf]

Supplemental materials

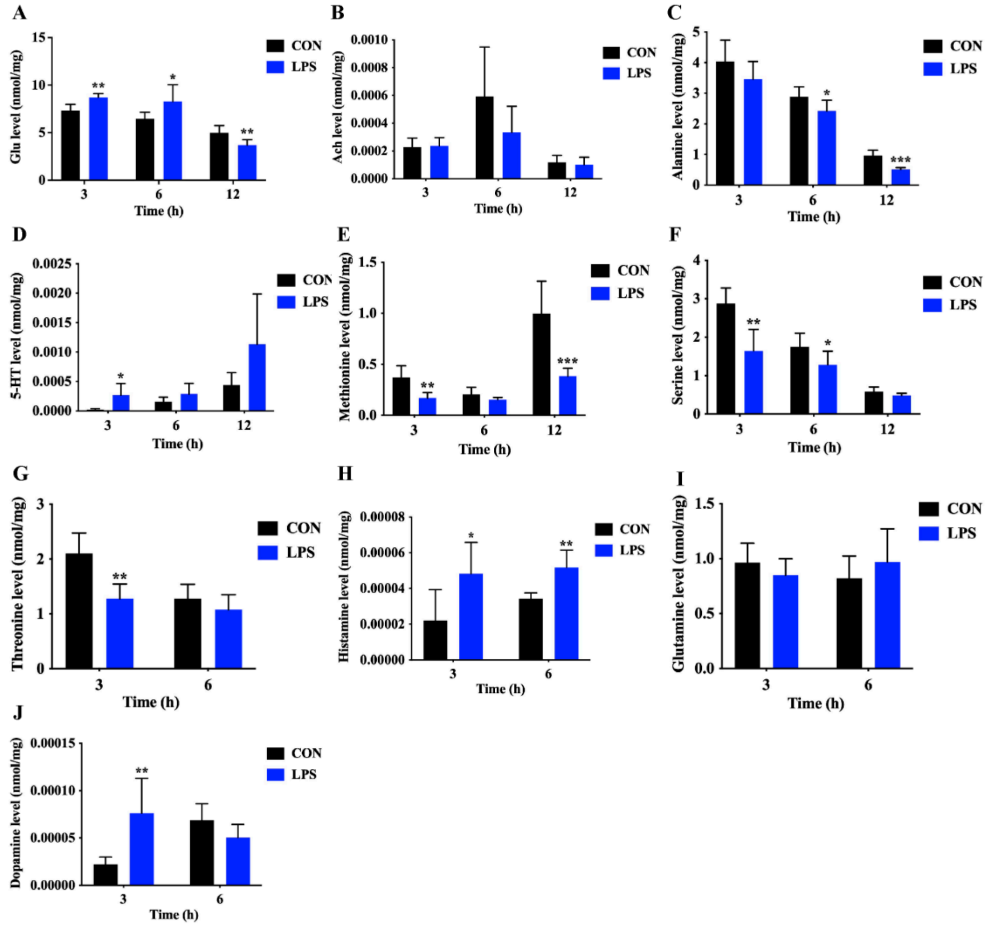

Supplementary Figure S1. Neurotransmitter levels in ileal tissue at different time points. (**A-J**) UHPLC-MS/MS of Glu, Ach, alanine, 5-HT, methionine, serine, threonine, histamine, glutamine and dopamine at different time points**.** Data are shown as mean ± SD. * indicates that the difference between the CON and the LPS is significant ($P < 0.05$). ** or *** indicates that the difference between the CON and the LPS is extremely significant ($P < 0.01$ or $P < 0.001$).

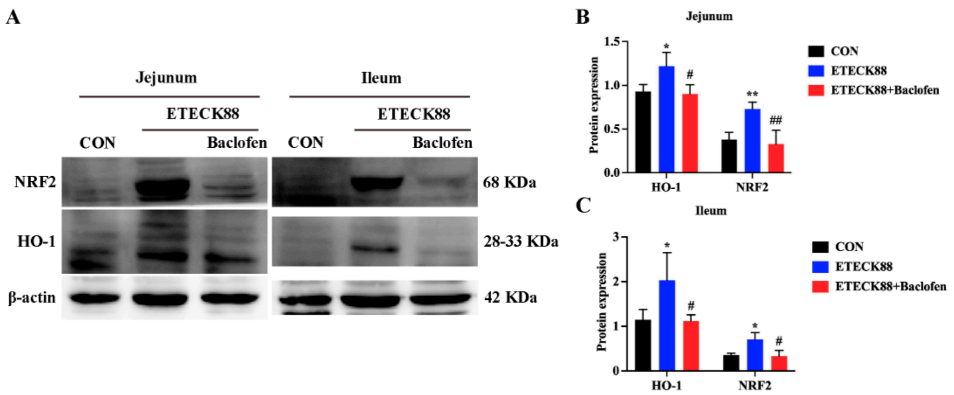

Supplementary Figure S2. GABA$_B$R activation could reduce oxidative stress in ETECK88-induced mice. (**A-C**) WB analysis the expression of NRF2 and HO-1 in ileal tissues of mice. For WB, expression levels were normalized to the expression of β-actin. Data are shown as mean ± SEM. * indicates that the difference between the CON and the ETECK88 is significant (*P* < 0.05). ** indicates that the difference between the CON and the ETECK88 is extremely significant (*P* < 0.01). # indicates that the difference between the ETECK88 and the ETECK88 + Baclofen is significant (*P* < 0.05). ## indicates that the difference between the ETECK88 and the ETECK88 + Baclofen is extremely significant (*P* < 0.01).
